# Microstructural Analysis of Termite Wings: Implications for Hydrophobic Adaptations in Rainy Flight

**DOI:** 10.3390/insects17040393

**Published:** 2026-04-03

**Authors:** Yongheng Shen, Ziheng Xue, Xuecheng Zhang

**Affiliations:** 1 Yuexi Campus, Suzhou Institute of Industrial Technology, Suzhou 215100, China; 2 Department of Life Sciences, Imperial College London, London SW7 2AZ, UK; 3 Jiangsu Lingte Technology Co., Ltd., Xuzhou 221000, China; xueziheng44@gmail.com; 4 Institute of Western Agriculture, Chinese Academy of Agricultural Sciences, Changji City 831100, China; xuecheng111@outlook.com

**Keywords:** termite wings, hydrophobic adaptation, microstructural analysis, flight in rain, setae and micrasters

## Abstract

Many termite species initiate colonization flights during or shortly after periods of rain, employing two different flight strategies: flying during the day in the rain and flying at night in a dry environment. As noted in previous studies, it appears easier for a species to become adapted to a wet environment by changing the contour/shape rather than the composition of the cuticle surface. We utilized differential interference contrast and scanning electron microscopy to observe the micro-nanostructure of the wing cuticles of 54 termite species from 16 families/subfamilies. Twenty-four species of higher termites possessed wings with anti-wetting structures of setae and a micraster array. The majority of lower termite wings had smoother cuticle surfaces. Based on the hierarchical design of termite wings, we conclude that various species are adapted to flying in the rain.

## Simple Summary

Termites reproduce by annual dispersal and usually choose to fly during or after the rainy season when soil conditions are favourable for them to dig and establish new homes. According to previous studies, the adaptation of termites to rainwater is reflected in the microstructures of their wings. At present, the colonization flight strategies of other termite species during the rainy season remain uncertain, except for the two confirmed hydrophobic and one hydrophilic termite genera. In this study, we used microscopy to observe the microstructure of wings of 54 different termite species from 16 families/subfamilies. It was found that most of the higher termite wings are suitable for flying in the rain, while wings of lower termites are better suited for flight in dry conditions.

## 1. Introduction

Insects owe much of their evolutionary success to flight [1]. Flying insects can avoid predators, search for food, and colonize new environments better than their flightless relatives [2,3]. The timing and environmental setting of flight are the major determinants of fitness and the insect’s ecological role [4,5,6]. Rainfall as an environmental factor can affect the performance of insect flight; the high impact pressure of falling water droplets can damage the microstructure of the wings [7,8]. In the majority of insects, wing adaptations to rain during flight are structural rather than chemical [9].

Termite colonies reproduce through annual dispersal. This is a seasonal phenomenon in which alates find mates at new nesting sites by flying away from the original colony [10]. Many termite species typically fly during or shortly after the rainy season, partially because in many ecosystems the ground is only soft enough during the rainy season for alates to dig into and establish new nests [10,11]. The two flight strategies employed by termite species are flying in the rain during the day and flying in dry conditions at night. The flight strategies have corresponding differences in the microstructure of the wings [12]. The two currently known hydrophobic termite genera (*Nasutitermes* sp. and *Microcerotermes* sp.) exhibit a hierarchical array structure in the wing cuticle that minimizes the interaction of the wing cuticle with water droplets through a combination of setae arrays and star-shaped micrasters [11]. The hydrophilic termite genus *Schedorhinotermes* shows smaller-scale folds and ridges in a hexagonal arrangement in the wing cuticle and the absence of setae and micraster arrays, making it easier for water droplets to spread on the wing [11,13].

At present, the colonization flight strategies of other termite species during the rainy season remain uncertain, except for the two confirmed hydrophobic and one hydrophilic termite genera. Watson et al. examined the hydrophilic and hydrophobic surface structures of termite wings and demonstrated that the wings exhibited the surface-wetting property of dual wettability [11,13,14]. In this study, differential interference contrast (DIC) microscopy and scanning electron microscopy (SEM) were used to observe the microstructures of the wing cuticle of 24 species of higher termites from 10 subfamilies and 42 species of lower termites from 10 families to classify the hydrophobicity of their wings and predict the potential for flight in the rain.

## 2. Materials and Methods

### 2.1. Sample Collection

The termite specimens were obtained from the Natural History Museum in London, which has one of the largest collections of termites in the world and holds over half of the known termite species on Earth.

### 2.2. Differential Interference Contrast Microscopy

A BX63 DIC microscope (Olympus Corporation, Tokyo, Japan), which provides three-dimensional images, was used for microscale observations (Table 1). The termite body was held with blunt tweezers, and the wing was removed with sharp tweezers. The separated wing was dried using lens tissue, and any remaining ethanol was rinsed off with Euparal gel on a spare slide glass before placing the wing onto the final slide. The wings were imaged at 50 μm and 20 μm using 10×, 20×, and 40× objective lenses. The Cellsens Dimension software (V4.1.1, Evident company, Tokyo, Japan) automatically generates recommended step sizes based on the focal length scale of the image and calculates the number of images to be captured. Finally, images taken at different focal length scales are then rendered into a single clean image by the Helicon software (V8.2.2, HeliconSoft company, Kharkiv, Ukraine).

### 2.3. Scanning Electron Microscopy

After microscale imaging, 11 species (*Tuberculitermes guineensis*, *Macrotermes michaelseni*, *Schedorhinotermes*, *Prorhinotermes inopinatus*, *Coptotermes acinaciformi*, *Cryptotermes brevis*, *Postelectrotermes militaris*, *Bicornitermes bicornis*, *Mastotermes darwiniensis*, *Microhodotermes viator*, and *Hodotermes mossambicus*) were selected for nanoscale observation. Before the SEM, the wing samples were immersed in a graded series of ethanol concentrations for preliminary dehydration, gradually transferring the wing samples to 85% and 95% ethanol for 20 min, then transferred to absolute ethanol (100%) for 30 min. The wing specimens were placed in a Leica EM CPD300 for critical-point drying (Vienna, Austria). After dehydration, the specimens were fixed on aluminum stubs. Since the termite wing specimens were very thin and light, they tended to curl after drying; therefore, carbon conductive tabs were placed on the aluminum stubs in advance. After metal coating, a Zeiss Ultra Plus Field Emission (SEM) (Oberkochen, Germany) was used to image the wing specimens.

## 3. Results

### 3.1. Hierarchical Structure

**Figure 1:**
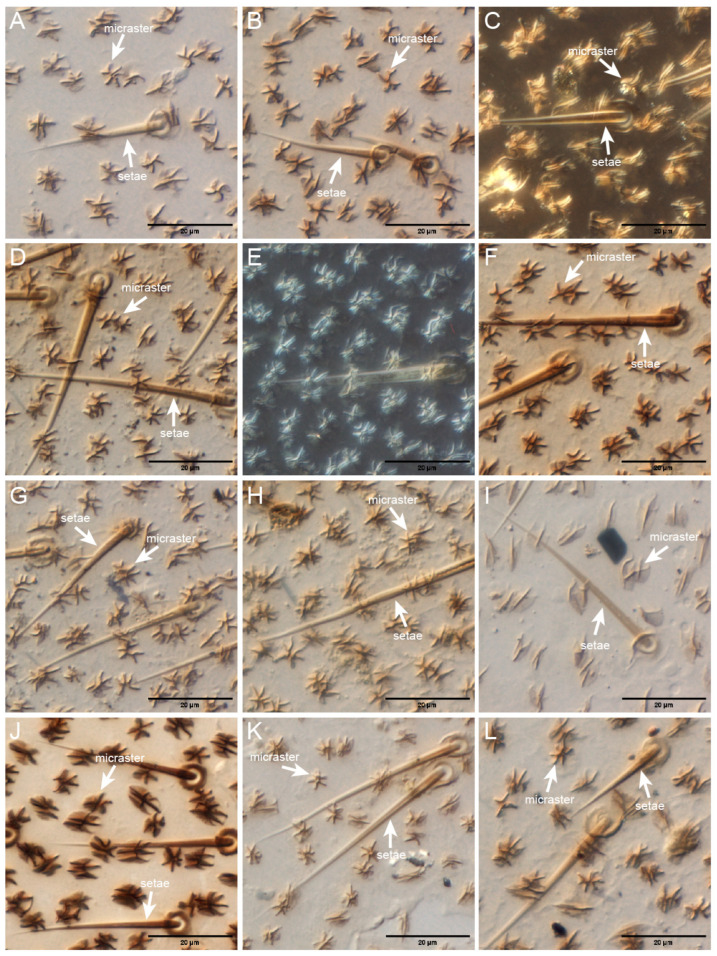
Anti-wetting hierarchical arrangement of the setae/micrasters. (**A**) *Microcerotermes zuluensis*; (**B**) *Microcerotermes parvus*; (**C**) *Euchilotermes tensus*; (**D**) *Cephalotermes rectangularis*; (**E**) *Nasutitermes novarumhebridarum*; (**F**) *Nasutitermes arborum*; (**G**) *Nasutitermes banksi*; (**H**) *Nasutitermes costalis*; (**I**) *Spinitermes trispinosus*; (**J**) *Termes hospes*; (**K**) *Termitogeton planus*; (**L**) *Microcerotermes turneri*.

Of the species observed, 22 had setae and micraster arrays, 20 species of higher termites and 2 species of lower termites (Table 2). The density of setae varied among species. *Microcerotermes zuluensis*, *Microcerotermes parvus*, *Euchilotermes tensus*, *Cephalotermes rectangularis*, *Nasutitermes novarumhebridarum*, *Nasutitermes arborum*, *Nasutitermes banksi*, *Nasutitermes costalis*, *Spinitermes trispinosus*, *Termes hospes*, *Termitogeton planus* and *Microcerotermes turneri* had a higher setae density, an even distribution of micraster and setae arrays, and showed a large-scale roughness on the wing surface (Figure 1).

### 3.2. Star-Shaped Micraster Structure

**Figure 2:**
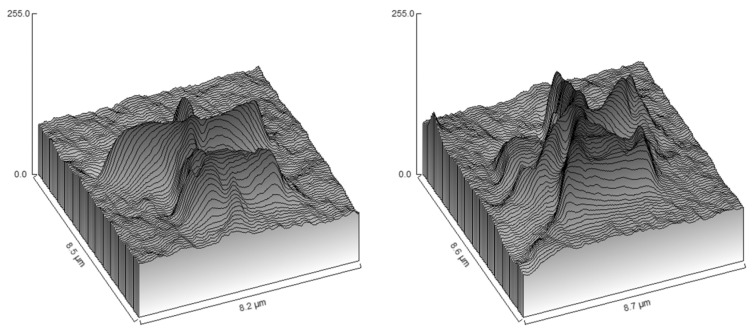
Micraster Geometry of *Microcerotermes zuluensis* by Surface Plot.

The smaller-scale structures that exist between the setae on the wing cuticle are called micrasters. The structures of the micrasters have been examined in several termite species [15,16,17]. These star-shaped structures consist of 5–6 arms, with the width (extremity-to-extremity distance) ranging from 5 to 8 μm (Figure 2). Previous studies have confirmed that this cluster structure can support small water droplets, causing the droplets to form a Cassie-Baxter state of interaction [11].

### 3.3. The Array Structure of Double-Layered Setae

**Figure 3:**
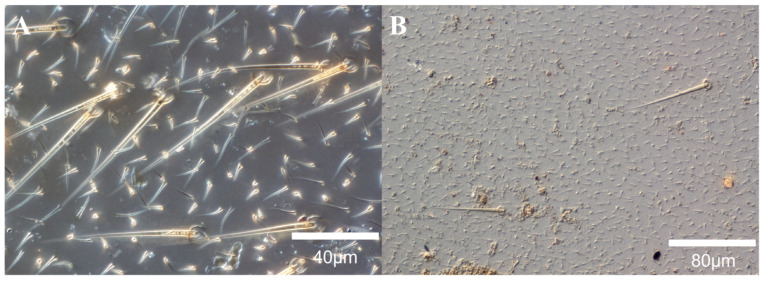
Morphology of double-layer setae structure. (**A**) *Eutermellus convergens*; (**B**) *Sphaerotermes sphaerothorax*.

The wings of *Eutermellus convergens* and *Sphaerotermes sphaerothorax* possessed a unique double-layered setae structure without micrasters. The length of the long setae array of *Eutermellus convergens* was 70–75 μm, and the length of the short setae array was 10–20 μm (Figure 3A). *Sphaerotermes sphaerothorax* had sparsely distributed long setae, with intervals between the setae reaching 200 μm or more; the length of short setae arrays was 5–10 μm (Figure 3B).

### 3.4. Species with a Setae Array Without Micrasters

**Figure 4:**
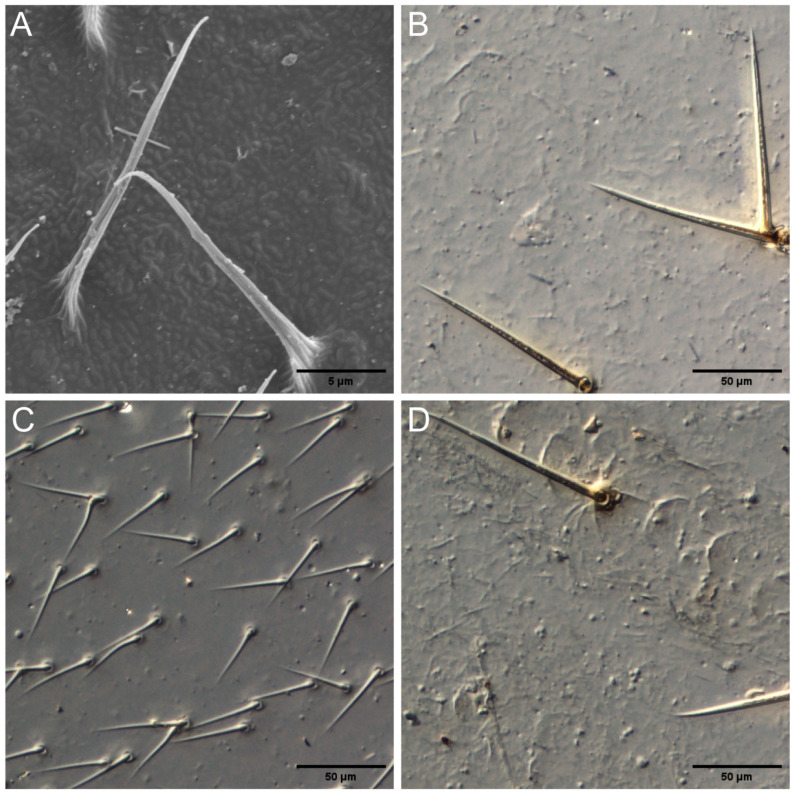
Morphology of setae array. (**A**) *Tuberculitermes guineensis* (×1046); (**B**) *Macrotermes michaelseni*; (**C**) *Coptotermes acinaciformis*; (**D**) *Postelectrotermes militaris*.

*Tuberculitermes guineensis* and *Postelectrotermes militaris* had longer arrays of setae on the wing veins; the setae were sparsely distributed and no microsculpture structures were observed (Figure 4A,D). *Macrotermes michaelseni* setae were concentrated on the costal margin (Figure 4B). *Coptotermes acinaciformis* had a smooth surface with densely distributed setae (Figure 4C).

### 3.5. No Setae or Micrasters

**Figure 5:**
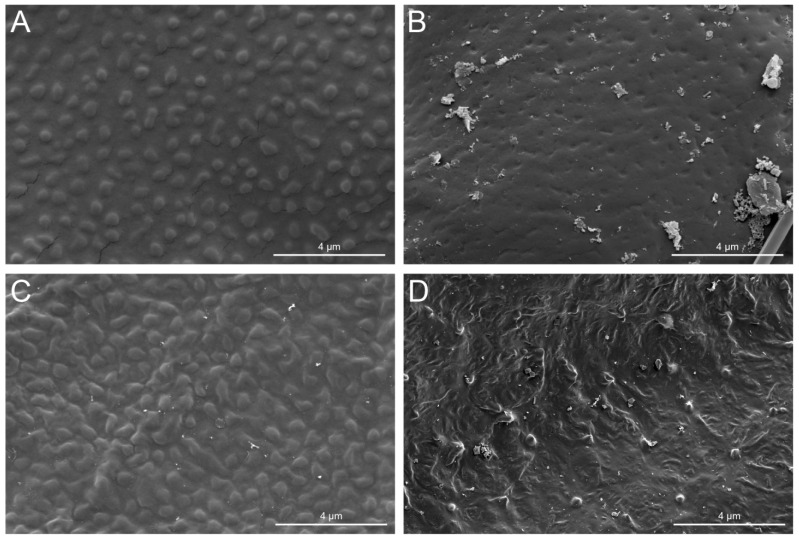
Morphology of termite wings without significant structure. (**A**) *Schedorhinotermes Holmgreni* (×5000); (**B**) *Mastotermes darwiniensis* (×5000); (**C**) *Bicornitermes bicornis* (×4730); (**D**) *Hodotermes mossambicus* (×500).

No significant structures were observed on the wing surfaces of 38 termite species (Table 2), with the wings showing only nano-sized curved projections and smooth surfaces (Figure 5).

### 3.6. Microstructure and Flight Strategy

To facilitate cross-taxon comparison, a summary table is provided that maps each species to its corresponding structural type and inferred flight strategy (Table 3). This table synthesizes the key relationships identified in our analysis, highlighting the association between morphological variation and flight modes.

## 4. Discussion

### 4.1. Hydrophobic Structures Associated with Insect Taxonomy

Termites are classified as higher and lower species. Higher termites are those in the family Termitidae, and lower termites comprise all remaining families [18]. The criteria for classification are primarily based on diet and digestion. Lower termites typically feed on wood and rely on gut protist symbionts to digest lignocellulose, while higher termites have a wider range of food sources, including wood, grasses, dead leaves, epiphytes, and organic and inorganic components of soils. Higher termite digestion is due to the loss of the protists and the acquisition of more complex gut structures with symbiotic associations with fungi and bacteria [19].

Micron-sized setae and micraster structures (arrays) are commonly found on the wings of higher termites. All higher termites observed in this study had micron-sized setae. *Sphaerotermes sphaerothorax* (Sphaerotermitinae) and *Eutermellus convergens* (Nasutitermitinae: Subulitermitini) had micron-sized double-layered setae structures. This unique setae structure accounted for 7% of all observed higher termite samples. Four species of lower termites had clear setae structures on their wing surfaces, and *Stolotermes ruficeps* (Termopsidae) and *Termitogeton planus* (Rhinotermitidae) had both setae structures and micraster arrays. Micro- and nano-scale roughness on the insect cuticle reduces surface adhesion by decreasing the contact area with the external environment (Figure 1 and Figure 2) [20]. Most higher termites have micrasters/microsculpture structures on their wings, but no micraster arrays were observed in four species of higher termites (*Tuberculitermes guineensis, Macrotermes michaelseni, Sphaerotermes sphaerothorax* and *Eutermellus convergens*), accounting for 14.8% of the sampled higher termites.

### 4.2. The Unique Hydrophobic Structure of Termite Wings

The colonial flight of termites refers to night flying in dry conditions and daylight flight in the rain (to avoid predators). Termites are one of the few insects that can successfully fly in the rain due to the unique hydrophobic structure of the wing surface [13]. Termites have a very high wing surface area/body mass (SA/M) ratio, and insects with a high SA/M usually have waterproof wings [11]. Termites usually fly for short periods and distances. Wing flutter is not a determining factor for termite flight in the rain, as the wing flapping rate is low, and the timing of the interaction of the cuticle on the wing surface with water droplets is more important for maintaining controlled flight [10,21]. Therefore, the special hydrophobic structure of the termite wing cuticle is crucial to the success of termite flight in rain [11]. Research on self-cleaning materials based on molecular architecture has demonstrated that superhydrophobic surfaces are typically rough, with a layered nano/micron structural morphology [22]. Watson et al. tested the effect of the layered design on the wings of two species of hydrophobic termites using water droplet experiments and contact angle calculations. Their results showed that this unique setae/micraster structure acts as a support for water droplets, accelerating their removal from the wings during flight in the rain [13,23]. The hierarchical design of the hydrophobic termite wing cuticle refers to the array of setae combined with the micraster array [24]. Setae are used to hold larger droplets, and micrasters are used to reduce the contact between the micro-droplets and the wing cuticle [25]. A contact angle greater than 90 degrees means that the water droplets will have a lower affinity for the wing surface. The liquid water will take much longer to diffuse across the wing than in the setaeless, micraster-free wings of hydrophilic termites, and the droplets can be more easily shed [26,27]. The micraster structure of the termite wing typically consists of 5–7 arms, most of which show micron-sized, star-shaped structures, with setae arrays and micraster structures at the micrometre scale. Compared to those of hydrophobic termites, the wing surfaces of hydrophilic termites (e.g., Schedorhinotermes) are without arrays of setae or micrasters, showing hexagonal ridges spaced at 600–900 nm intervals under scanning electron microscope observation. A study of the nanoscale hexagonal arrays on the wings of termites suggested that the “corneal nipple arrays” reflect light in the visible spectrum as a strategy for avoiding predators [28]. According to the research on the wings of termites (family Rhinotermitidae) and cicadas (*Pflatoda claripennis*), the cross-sectional area of termite wings is only about one-eighth that of cicadas [29,30]. The effective Young’s modulus of the wing sections is inversely proportional to the third power of the thickness; thus, termite wings show high expected stiffness [31]. Nanoscale arrays on the wing surfaces of hydrophilic termites (Schedorhinotermes) may also play a role in maintaining flight stability [11,30].

### 4.3. Loss of Superhydrophobicity and the Role of Surface Materials

An important observation in this study is that some specimens, particularly those preserved for long periods in museum collections, had lost their superhydrophobicity. This raises questions regarding the stability and material basis of the observed surface structures. One possible explanation is that the loss of hydrophobicity results from damage, degradation, or removal of surface nanostructures over time, particularly under long-term storage in ethanol. Recent work by Rebora et al. on mayfly wings demonstrated that 10 days after insect death, wax crystals on the wing surface tend to change their shape and become flattened structures over the cuticle [32]. In that study, the degradation of wax-based nanostructures led to significant changes in surface properties, including reduced hydrophobicity and altered optical behavior [32].

This finding is particularly relevant to the present study because the SEM images (Figure 5B,D) show partially damaged or reduced surface structures rather than completely smooth cuticles, which is similar to the degradation patterns observed in mayfly wings. Therefore, caution is needed when interpreting the absence or reduction of structures in preserved specimens. The use of museum material represents an inherent limitation of the study, as structural degradation may lead to underestimation of the complexity or functionality of the original wing surfaces. Nevertheless, the clear phylogenetic signal observed across multiple taxa supports the robustness of the main conclusions.

### 4.4. Hydrophobic Structures in a Broader Biological and Biomimetic Context

Hydrophobic and superhydrophobic surface structures are widespread in biological systems and have attracted considerable attention in both evolutionary biology and biomimetic engineering. Research by Finet et al. and Gomez et al. on wing transparency and hydrophobicity indicates that microstructural and nanostructural diversity plays a crucial role in balancing optical transparency and water repellency in Lepidoptera [33,34]. For example, contact angles were measured on the black-scaled and transparent region of the wings of *Phanus vitreus* (clearwing butterfly) to assess their hydrophobicity [33]. These studies highlight that structural features such as scale reduction, nanopillar arrays, and hierarchical roughness can be gained or lost during evolution, leading to trade-offs between visibility, wettability, and ecological function. This evolutionary plasticity is consistent with the patterns observed in this study, where higher termites exhibit complex hierarchical structures (setae + micrasters), whereas lower termites tend to show a smoother hydrophilic wing surface. These findings suggest that termite wing structures should be interpreted not as isolated adaptations, but as part of a broader continuum of biological surface designs in which micro- and nanostructures are tuned to meet multiple functional demands, including hydrophobicity, self-cleaning, and potentially optical performance.

Beyond the specialized micro- and nanostructures observed on insect wings, cuticular adaptations contributing to hydrophobicity are widespread across diverse body regions and taxa. In particular, the density and morphology of setae on insect legs play a critical role in modulating surface wettability and water repellency [35]. For example, many semi-aquatic and terrestrial insects possess dense arrays of hydrophobic setae that trap air and reduce contact with water, thereby enhancing their ability to remain dry and mobile in wet environments [36].In aquatic insects, similar principles are observed in the formation of plastrons—thin layers of air retained by dense cuticular hairs—which function as physical gills and contribute to both respiration and hydrophobic performance [37,38]. These structures demonstrate that hydrophobicity in insects is not limited to wing surfaces but represents a generalized functional trait mediated by hierarchical cuticular architecture across multiple regions. Such diversity highlights the evolutionary significance of microstructured surfaces in enabling insects to exploit a wide range of ecological niches.

### 4.5. Adaptive Strategies for Rain-Based Dispersal

We predicted the ability of several species of termites to fly in the rain based on the dual wettability of their wings. All higher termites had setae arrays. The nanostructures of two species with only setae and no micrasters were observed by scanning electron microscopy. The wing cuticles of *Tuberculitermes guineensis* and *Macrotermes michaelseni* had a special shape of ridges, but their roughness scale was very small (the array of hydrophobic micrasters measured 5–6 μm), which does not fit the hierarchical structure design of hydrophobic termite wings [11]. Setae arrays and micrasters were found for the remaining 13 higher termite species (Table 2), consistent with the hierarchical moisture-resistant structure found by Watson et al. *Coptotermes acinaciformis* had a wing structure similar to that of the hydrophobic termite, with a double-layered structure of the wing cuticle.

Colonial flights of adult termites are usually seasonal. Adult dispersal is influenced by both environmental and internal factors. The environmental factors include precipitation and predators [39]. Based on a study of the flight activity of *Macrotermes gilvus* and *Macrotermes Carbonarius*, *Macrotermes gilvus* spreads its flights out over a long period before dawn (1–2.5 h) in the form of multiple swarms, thereby receiving less attention from predators [40,41]. *Macrotermes carbonarius* tends to operate large colonisation flights before the rainy season [41]. The large number of alates produced is probably an anti-predator, herd-like defence [42]. During one observational study, termites had a high probability of being captured by predators [43]. For example, a white-browed sparrow weaver (*Plocepasser mahali*) consumed 19 alates, whereas a hamerkop (*Scopus umbretta*) took 47 alates in a 5-min period [43]. Thus, the ability of many termite species to fly in the rain during the day or at night after a rain may be an adaptive strategy.

This pattern is consistent with the broader evolutionary framework proposed in recent studies of insect surface structures, where gains and losses of micro- and nanostructures reflect ecological trade-offs rather than linear optimization. As shown in Lepidoptera, structural simplification or reduction can be advantageous when transparency or other functions are prioritized, whereas increased structural complexity may evolve under selective pressures such as water exposure or contamination [34].

## 5. Conclusions

In this study, we examined the microstructure of the wing cuticles of 54 termite species from 16 families/subfamilies, with a focus on the hydrophobic adaptations of termites that fly in the rain. The research revealed that the wings of higher termites (24 species) typically featured anti-wetting structures, such as setae or micrasters, on the wing cuticle, whereas the wing cuticles of most lower termites were smooth. This structural difference affects the hydrophilic and hydrophobic properties of the wings, explaining the divergence in rainy flight strategies among different termite species. The hierarchical micro-nanostructure of termite wings is a key factor in their ability to adapt to flying in the rain. For example, hydrophobic termites (e.g., *Nasutitermes* sp. and *Microcerotermes* sp.) effectively reduce the contact between water droplets and the wings through arrays of setae and micrasters, while hydrophilic termites (e.g., *Schedorhinotermes* sp.) lack such structures, resulting in water droplets easily wetting their wings. These findings not only verify previous studies on the hydrophobicity of termite wings but also expand our understanding of the mechanisms of flight adaptation of other termite species. The results of this study provide a new perspective for understanding the relationship between termite flight and environmental adaptation.

## Figures and Tables

**Table 1:** Species of termite specimens and imaging numbers used by DIC microscopy.

Categories	Family	Subfamily	Infrafamily	Species	Number of Wing Specimens	Number of DIC Images
Higher termites	Termitidae	Amitermitinae	Amitermitini	*Microcerotermes subtilis*	3	3
				*Microcerotermes solidus*	3	3
				*Microcerotermes turneri*	4	11
				*Microcerotermes zuluensis*	4	9
				*Microcerotermes parvus*	3	6
				*Microcerotermes parvulus*	2	4
				*Microcerotermes strunckii*	3	4
		Cubitermitinae	Cubitermitini	*Cubitermes glebae*	7	9
		Macrotermitinae		*Macrotermes michaelseni*	3	7
		Nasutitermitinae	Nasutitermitini	*Nasutitermes novarumhebridarum*	3	13
				*Nasutitermes arborum*	3	8
				*Nasutitermes banksi*	3	8
				*Nasutitermes costalis*	3	9
			Subulitermitini	*Eutermellus convergens*	3	12
		Protohamitermitinae		*Protohamitermes globiceps*	3	13
		Sphaerotermitinae		*Sphaerotermes sphaerothorax*	4	12
		Termitinae	Capritermitini	*Capritermes capricornis*	6	18
			Microcapritermitini	*Mirocapritermes connectens*	3	10
			Spinitermitini	*Spinitermes trispinosus*	3	7
			Termitini	*Termes hospes*	3	10
Lower termites	Rhinotermitidae			*Reticulitermes lucifugus*	3	9
				*Schedorhinotermes Holmgreni*	3	7
				*Coptotermes acinaciformis*	2	7
				*Termitogeton planus*	3	12
				*Prorhinotermes inopinatus*	2	6
				*Psammotermes hybostoma*	2	2
	Serritermitidae			*Lack of available alates*		
	Stylotermitidae			*Lack of available alates*		
	Kalotermitidae			*Kalotermes flavicollis*	3	3
				*Bifiditermes durbanensis*	3	6
				*Bifiditermes mutabae*	2	3
				*Paraneotermes simplicornis*	3	5
				*Pterotermes occidentis*	3	5
				*Marginitermes hubbardi*	6	5
				*Ceratokalotermes spoliator*	2	6
				*Glyptotermes ueleensis*	1	2
				*Glyptotermes brevicaudatus*	4	6
				*Rugitermes rugosus*	1	6
				*Incisitermes milleri*	2	6
				*Postelectrotermes militaris*	2	4
				*Proneotermes perezi*	1	2
				*Neotermes arburiensis*	2	4
				*Neotermes camerunensis*	1	3
				*Neotermes sanctaecrucis*	1	3
	Hodotermitidae			*Anacanthotermes vagans*	3	9
				*Microhodotermes viator*	2	5
	Archotermopsidae			*Archotermopsis wroughtoni*	2	5
				*Zootermopsis angusticollis*	3	8
	Mastotermidae			*Mastotermes darwiniensis*	3	8
Total					144	344

**Table 2:** The distribution of setae and micrasters of higher and lower termites.

Higher Termites				
Categories	Family	Subfamily	Infrafamily	Species
With Setae and Micrasters Arrays	Termitidae	Amitermitinae	Amitermitini	*Microcerotermes subtilis*
				*Microcerotermes solidus*
				*Microcerotermes turneri*
				*Microcerotermes zuluensis*
				*Microcerotermes parvus*
				*Microcerotermes parvulus*
				*Microcerotermes strunckii*
		Cubitermitinae	Cubitermitini	*Cubitermes glebae*
			Basidentitermitini	*Orthotermes depressifrons*
			Euchilotermitini	*Euchilotermes tensus*
		Cylindrotermitinae		*Cephalotermes rectangularis*
		Nasutitermitinae	Nasutitermitini	*Nasutitermes novarumhebridarum*
				*Nasutitermes arborum*
				*Nasutitermes banksi*
				*Nasutitermes costalis*
		Protohamitermitinae		*Protohamitermes globiceps*
		Termitinae	Capritermitini	*Capritermes capricornis*
			Microcapritermitini	*Mirocapritermes connectens*
			Spinitermitini	*Spinitermes trispinosus*
			Termitini	*Termes hospes*
With Double-layered Setae without Micrasters Arrays		Nasutitermitinae	Subulitermitini	*Eutermellus convergens*
		Sphaerotermitinae		*Sphaerotermes sphaerothorax*
With Setae without Micrasters Arrays		Macrotermitinae		*Macrotermes michaelseni*
		Cubitermitinae	Tuberculitermitini	*Tuberculitermes guineensis*
**Lower termites**				
Without Setae and Micrasters Arrays	Rhinotermitidae			*Prorhinotermes inopinatus*
				*Psammotermes hybostoma*
				*Reticulitermes lucifugus*
				*Schedorhinotermes intermedius actuosus Hill*
				*Schedorhinotermes intermedius Brauer*
				*Schedorhinotermes Holmgreni*
	Archotermopsidae			*Zootermopsis angusticollis*
				*Archotermopsis wroughtoni*
	Termopsidae			*Porotermes adamsoni*
				*Porotermes planiceps*
	Hodotermitidae			*Anacanthotermes vagans*
				*Anacanthotermes ochraceus*
				*Microhodotermes viator*
	Kalotermitidae			*Bicornitermes bicornis*
				*Bifiditermes durbanensis*
				*Bifiditermes mutabae*
				*Bifiditermes sibayensis*
				*Calcaritermes nigriceps*
				*Ceratokalotermes spoliator*
				*Comatermes perfectus*
				*Cryptotermes brevis*
				*Cryptotermes domesticus*
				*Eucryptotermes wheeleri*
				*Epicalotermes kempae*
				*Glyptotermes brevicaudatus*
				*Glyptotermes ueleensis*
				*Incisitermes milleri*
				*Kalotermes flavicollis*
				*Marginitermes hubbardi*
				*Neotermes arburiensis*
				*Neotermes camerunensis*
				*Neotermes sanctaecrucis*
				*Paraneotermes simplicornis*
				*Proneotermes perezi*
				*Rugitermes rugosus*
				*Pterotermes occidentis*
				*Rugitermes nodulosus*
	Mastotermidae			*Mastotermes darwiniensis*
With Setae without Micrasters Arrays	Rhinotermitidae			*Coptotermes acinaciformis*
	Kalotermitidae			*Postelectrotermes militaris*
With Setae and Micrasters Arrays	Termopsidae			*Stolotermes ruficeps*
	Rhinotermitidae			*Termitogeton planus*

**Table 3:** Summary of Termite Species, Wing Microstructure and Inferred Flight Strategy.

Species	Structure Type	Inferred Flight Strategy
*Microcerotermes subtilis*	Setae and Micraster Arrays	Daytime flight in rain
*Microcerotermes solidus*	Setae and Micraster Arrays	Daytime flight in rain
*Microcerotermes turneri*	Setae and Micraster Arrays	Daytime flight in rain
*Microcerotermes zuluensis*	Setae and Micraster Arrays	Daytime flight in rain
*Microcerotermes parvus*	Setae and Micraster Arrays	Daytime flight in rain
*Microcerotermes parvulus*	Setae and Micraster Arrays	Daytime flight in rain
*Microcerotermes strunckii*	Setae and Micraster Arrays	Daytime flight in rain
*Cubitermes glebae*	Setae and Micraster Arrays	Daytime flight in rain
*Orthotermes depressifrons*	Setae and Micraster Arrays	Daytime flight in rain
*Euchilotermes tensus*	Setae and Micraster Arrays	Daytime flight in rain
*Cephalotermes rectangularis*	Setae and Micraster Arrays	Daytime flight in rain
*Nasutitermes novarumhebridarum*	Setae and Micraster Arrays	Daytime flight in rain
*Nasutitermes arborum*	Setae and Micraster Arrays	Daytime flight in rain
*Nasutitermes banksi*	Setae and Micraster Arrays	Daytime flight in rain
*Nasutitermes costalis*	Setae and Micraster Arrays	Daytime flight in rain
*Protohamitermes globiceps*	Setae and Micraster Arrays	Daytime flight in rain
*Capritermes capricornis*	Setae and Micraster Arrays	Daytime flight in rain
*Mirocapritermes connectens*	Setae and Micraster Arrays	Daytime flight in rain
*Spinitermes trispinosus*	Setae and Micraster Arrays	Daytime flight in rain
*Termes hospes*	Setae and Micraster Arrays	Daytime flight in rain
*Stolotermes ruficeps*	Setae and Micraster Arrays	Daytime flight in rain
*Termitogeton planus*	Setae and Micraster Arrays	Daytime flight in rain
*Eutermellus convergens*	Double-layered Setae Structure	Daytime flight in rain
*Sphaerotermes sphaerothorax*	Double-layered Setae Structure	Daytime flight in rain
*Macrotermes michaelseni*	Setae Array Only (No Micrasters)	Nighttime flight in dry conditions
*Tuberculitermes guineensis*	Setae Array Only (No Micrasters)	Nighttime flight in dry conditions
*Coptotermes acinaciformis*	Setae Array Only (No Micrasters)	Nighttime flight in dry conditions
*Postelectrotermes militaris*	Setae Array Only (No Micrasters)	Nighttime flight in dry conditions
*Prorhinotermes inopinatus*	No Setae or Micrasters (Smooth Surface)	Nighttime flight in dry conditions
*Psammotermes hybostoma*	No Setae or Micrasters (Smooth Surface)	Nighttime flight in dry conditions
*Reticulitermes lucifugus*	No Setae or Micrasters (Smooth Surface)	Nighttime flight in dry conditions
*Schedorhinotermes intermedius actuosus Hill*	No Setae or Micrasters (Smooth Surface)	Nighttime flight in dry conditions
*Schedorhinotermes intermedius Brauer*	No Setae or Micrasters (Smooth Surface)	Nighttime flight in dry conditions
*Schedorhinotermes Holmgreni*	No Setae or Micrasters (Smooth Surface)	Nighttime flight in dry conditions
*Zootermopsis angusticollis*	No Setae or Micrasters (Smooth Surface)	Nighttime flight in dry conditions
*Archotermopsis wroughtoni*	No Setae or Micrasters (Smooth Surface)	Nighttime flight in dry conditions
*Porotermes adamsoni*	No Setae or Micrasters (Smooth Surface)	Nighttime flight in dry conditions
*Porotermes planiceps*	No Setae or Micrasters (Smooth Surface)	Nighttime flight in dry conditions
*Anacanthotermes vagans*	No Setae or Micrasters (Smooth Surface)	Nighttime flight in dry conditions
*Anacanthotermes ochraceus*	No Setae or Micrasters (Smooth Surface)	Nighttime flight in dry conditions
*Microhodotermes viator*	No Setae or Micrasters (Smooth Surface)	Nighttime flight in dry conditions
*Bicornitermes bicornis*	No Setae or Micrasters (Smooth Surface)	Nighttime flight in dry conditions
*Bifiditermes durbanensis*	No Setae or Micrasters (Smooth Surface)	Nighttime flight in dry conditions
*Bifiditermes mutabae*	No Setae or Micrasters (Smooth Surface)	Nighttime flight in dry conditions
*Bifiditermes sibayensis*	No Setae or Micrasters (Smooth Surface)	Nighttime flight in dry conditions
*Calcaritermes nigriceps*	No Setae or Micrasters (Smooth Surface)	Nighttime flight in dry conditions
*Ceratokalotermes spoliator*	No Setae or Micrasters (Smooth Surface)	Nighttime flight in dry conditions
*Comatermes perfectus*	No Setae or Micrasters (Smooth Surface)	Nighttime flight in dry conditions
*Cryptotermes brevis*	No Setae or Micrasters (Smooth Surface)	Nighttime flight in dry conditions
*Cryptotermes domesticus*	No Setae or Micrasters (Smooth Surface)	Nighttime flight in dry conditions
*Eucryptotermes wheeleri*	No Setae or Micrasters (Smooth Surface)	Nighttime flight in dry conditions
*Epicalotermes kempae*	No Setae or Micrasters (Smooth Surface)	Nighttime flight in dry conditions
*Glyptotermes brevicaudatus*	No Setae or Micrasters (Smooth Surface)	Nighttime flight in dry conditions
*Glyptotermes ueleensis*	No Setae or Micrasters (Smooth Surface)	Nighttime flight in dry conditions
*Incisitermes milleri*	No Setae or Micrasters (Smooth Surface)	Nighttime flight in dry conditions
*Kalotermes flavicollis*	No Setae or Micrasters (Smooth Surface)	Nighttime flight in dry conditions
*Marginitermes hubbardi*	No Setae or Micrasters (Smooth Surface)	Nighttime flight in dry conditions
*Neotermes arburiensis*	No Setae or Micrasters (Smooth Surface)	Nighttime flight in dry conditions
*Neotermes camerunensis*	No Setae or Micrasters (Smooth Surface)	Nighttime flight in dry conditions
*Neotermes sanctaecrucis*	No Setae or Micrasters (Smooth Surface)	Nighttime flight in dry conditions
*Paraneotermes simplicornis*	No Setae or Micrasters (Smooth Surface)	Nighttime flight in dry conditions
*Proneotermes perezi*	No Setae or Micrasters (Smooth Surface)	Nighttime flight in dry conditions
*Rugitermes rugosus*	No Setae or Micrasters (Smooth Surface)	Nighttime flight in dry conditions
*Pterotermes occidentis*	No Setae or Micrasters (Smooth Surface)	Nighttime flight in dry conditions
*Rugitermes nodulosus*	No Setae or Micrasters (Smooth Surface)	Nighttime flight in dry conditions
*Mastotermes darwiniensis*	No Setae or Micrasters (Smooth Surface)	Nighttime flight in dry conditions

## Data Availability

The original contributions presented in this study are included in the article. Further inquiries can be directed to the corresponding author.
